# Supplementary material for: MetaRibo-Seq measures translation in microbiomes
Source: Nat Commun. 2020 Jun 29;11:3268. doi: 10.1038/s41467-020-17081-z (PMC7324362; doi:10.1038/s41467-020-17081-z)
Supplement: Supplementary file 10 — Supplementary Data 7 [file 41467_2020_17081_MOESM10_ESM.zip › File2/Confidence_VeryHigh_Taxonomy/11808_out.krona.html]

Javascript must be enabled to view this page.

members
magnitude
magnitudeUnassigned
count
unassigned
taxon
rank

11808\_out

18

superkingdom
15
2

phylum
1
976

class
1
200643

order
1
171549

1
815
family

1
816
genus

species
1897051

SRS142890\_contig\_number\_2827
1

1239
14
phylum

class
13
186801

13
186802
order

family
7
31979

genus
7
1485

7
1637498

SRS047014\_contig\_number\_1575SRS049959\_contig\_number\_1256SRS049959\_contig\_number\_44704SRS077294\_contig\_number\_8805SRS077294\_contig\_number\_contig-100\_2361.115428SRS142503\_contig\_number\_contig-100\_2001.93842SRS893252\_contig\_number\_contig-100\_20252.20253
species

family
541000
2

species
2

SRS023526\_contig\_number\_35374SRS049896\_contig\_number\_31012
1898205

family
216572
1

genus
459786
1

species
1

SRS065504\_contig\_number\_37139
1897012

family
186806
3

1730
3
genus

species
3
1262886

SRS015217\_contig\_number\_30884SRS015431\_contig\_number\_87843SRS019068\_contig\_number\_115481

species

SRS144183\_contig\_number\_40707
1263006
1

3

SRS048870\_contig\_number\_contig-100\_27302.71067SRS077392\_contig\_number\_41550SRS143895\_contig\_number\_31837
